# The Role of Transcatheter Aortic Valve Replacement in Asymptomatic Aortic Stenosis: A Feasibility Analysis

**DOI:** 10.7759/cureus.29522

**Published:** 2022-09-24

**Authors:** Muhammad Haseeb ul Rasool, Maleeha Saleem, Muhammad Nadeem, Mubasharah Maqbool, Ahmed Ali Aziz, Justin M Fox, Addi Suleiman

**Affiliations:** 1 Medicine, Icahn School of Medicine at Mount Sinai, Queens Hospital Center, New York, USA; 2 Internal Medicine, Saint Francis Medical Center, Trenton, USA; 3 Cardiology, Saint Michael's Medical Center, Newark, USA; 4 Internal Medicine, Tufts Medical Center, Medford, USA; 5 Cardiology, Saint Francis Medical Center, Trenton, USA

**Keywords:** clinical characteristics, asymtomatic, surgical aortic valve replacement (savr), tavr', aortic stenosis (as)

## Abstract

Surgical aortic valve replacement (SAVR) is the current treatment of choice for good surgical candidates with moderate to severe symptomatic aortic stenosis (AS). As transcatheter aortic valvular replacement (TAVR) has shown an improved one and two-year all-cause mortality, it has been chosen for moderately symptomatic severe AS patients. The purpose of this review was to perform a clinical comparison of TAVR vs. SAVR and to analyze the Health Index Factor (HIF) that makes TAVR a treatment of choice in asymptomatic AS patients.

An extensive literature search of PubMed, Cochrane, and Embase databases was performed using the keywords “Aortic stenosis”, “SAVR”, “TAVR”, and “Asymptomatic”. A total of 45 prospective randomized clinical trials in the English language that were published from the year 2000 onwards were included in the final analysis. It has been found that 59.3% of asymptomatic AS patients are likely to die in the next five years without proactive treatment. Multiple studies have proven that early intervention with aortic valve replacement is superior to conservative treatment in severe asymptomatic AS; however, the choice between SAVR and TAVR is not well established. The NOTION Trial, SURTAVI Trail, and PARTNER 3 study have shown the non-inferiority of TAVR over SAVR, during one-year follow-up for low surgical risk patients. Evolut Low-Risk study and Early TAVR are the only two prospective studies performed to date that have enrolled patients with asymptomatic severe AS. The Evolut Trial demonstrated no difference in all-cause mortality at 30 days (1.3% vs. 4.8%. p=0.23), and 12 days (1.3% vs. 6.5%, p=0.11). Additionally, TAVR also decreases the risk of post-procedural atrial fibrillation, acute kidney injury (AKI), and rehospitalization, and leads to significant improvement in the mean trans-aortic pressure gradient. TAVR also showed marked improvement in the 30-day Quality of Life (QOL) index, where SAVR did not report any significant change in the QOL index. However, the official recommendations of Early TAVR are still awaited. TAVR has consistently shown a statistically non-significant difference in case mortality, risk of stroke, and rehospitalization with moderate to high surgical risk patients whereby recent initial trials have shown significant improvement in the QOL index and hemodynamic index for patients with asymptomatic disease. More extensive studies are required to prove the risk stratifications, long-term outcomes, and clinical characteristics that would make TAVR a preferred intervention in asymptomatic patients.

## Introduction and background

Aortic stenosis (AS) is one of the most common acquired valvular heart conditions, the incidence of which increases with advancing age [1,2]. The 2014 American Heart Association (AHA) guidelines define the aortic jet velocity of at least 4.0 m/s or mean transvalvular gradient of at least 40 mmHg as severe AS [3]. The National Echo Database Australia (NEDA) has demonstrated that the incidence of AS increased from 5/1000 person-years at <30 years to 40/1000 person-years at age >80 years [4]. Half of the patients with severe AS lack any symptoms at the time of diagnosis [5,6]. There is a concern regarding an iceberg of undiagnosed cases of AS [7] that may lead to higher case-associated mortality once the patients become symptomatic [8]. Untreated patients of AS have mortality rates reaching up to 2% per month of the untreated period [9], which sums up to approximately 50% at two years and goes up to 97% at five years [10]. Despite the risk that a significant percentage of patients go on to develop congestive cardiac failure, pulmonary HTN, and myocardial fibrosis leading to death, there are no guidelines on the time and type of intervention in such patients [11].

It has been recommended that the patients who have an aortic jet velocity of at least 5 m/s, a mean trans-aortic pressure gradient of at least 60 mmHg, those developing a steep increase in aortic velocity (≥0.3 m/s per year), or those with LVEF ≤50 have an increased risk of sudden cardiac death. Additionally, patients with very severe AS having >5 m/s aortic jet velocity can present with acute coronary syndrome even with normal coronary vessels and are recommended to undergo aortic valve replacement on priority [5,12]. Aortic valve replacement, either transcatheter or surgical, has proven prognostic significance for symptomatic AS patients [2,13]. A systematic analysis conducted by Ullah et al. based on the results of eight studies involving 2201 patients documented that early intervention was associated with a reduction of all-cause mortality (OR: 0.24, 95% CI: 0.13-0.45, p<0.00001) and cardiovascular mortality (OR: 0.2, 95% CI: 0.06-0.70, p=0.01) as compared to conservative treatment. The number needed to treat (NNT) to prevent one case of all-cause and cardiovascular mortality was calculated as 4 (94% CI: 3.5-4.0) and 9 (95% CI: 7.0-9.0) respectively [14]. Similar results were found in a stratified analysis performed using the severity of AS approved as severe and very severe AS. Early intervention in the patients who were asymptomatic but had severe and very severe aortic stenosis resulted in improved overall mortality over an extended mean follow-up of 4.3 years (OR: 0.24, 95% CI: 0.11-0.52, p=0.0004 and OR: 0.20, 95% CI: 0.08-0.51, p=0.0008) [14]. In 2015, FDA also approved the use of TAVR for failed bio-prosthesis for the valve in valve procedure [15].

Currently, the clinical guidelines for asymptomatic patients recommend watchful waiting till the development of AS-related symptoms or LV systolic dysfunction [2,13]. Despite the demonstration of improved survival following TAVR in Partner 1 and Partner 2 trials [16,17], in the early 2010s, the ratio of patients who underwent TAVR was significantly low following the screening. Though the transfemoral approach is the most commonly used approach for TAVR with improved three-year post-procedure survival, it precludes the use of TAVR for patients with smaller iliofemoral arteries, and for those with peripheral artery disease. It wasn't until the latter half of the decade that the use of the trans-axillary and trans-caval approaches provided more feasible options for intervention [18,19,20,21]. We conducted an extensive literature search on PubMed, Cochrane, Google Scholar, and Clinicaltrials.gov using the MeSH terms “Aortic stenosis”, “SAVR”, “TAVR”, and “Asymptomatic`` to include all the studies conducted regarding treatment options for asymptomatic AS. This review intends to perform a feasibility analysis to describe the specific cohort of patients who, while being asymptomatic, would benefit from early intervention, and the intervention of choice should be TAVR over SAVR. This will benefit the healthcare professionals to support their decision based on evidence-based medicine and to promptly identify the group of patients who should readily be treated.

## Review

Disease burden of aortic stenosis

A contemporary population-based model based on 2019`s UK population presented by Strange et al. showed a point prevalence of severe AS at 1.48% in patients aged >55 years, which translates to 291448 cases. Of the total cases, 68.3% (199059, 95% CI: 177201-221355) were symptomatic, while rest (31.7%, 92389, 95% CI: 70093-144247) were asymptomatic. Out of the symptomatic cases, 58.4% (116251, 95% CI: 106895-125606) patients were eligible to undergo SAVR, out of which 7208 (95% CI: 7091-7234) carried high surgical risk. Whereas the remaining 41.6% (82809, 95% CI: 73453-92164) of symptomatic patients were unsuitable to undergo SAVR, 61.7% of these patients unfit for SAVR (51093, 95% CI: 34780-67655) underwent TAVR. It has also been proposed that out of the total 291448 people, 59.3% (172859) are prone to die in the next five years without proactive treatment, which far exceeds the local healthcare delivery system`s capacity. However, these statistics are based on several studies and population-based research, which cannot be completely verified for implementation in the general population. This presents the total disease burden in AS in the developed nations where the improved healthcare facilities are leading to an increased proportion of the aging population, which in turn can cause a further increase in the disease burden [7].

Risk stratification in aortic stenosis patients

Transcatheter aortic valve implantation (TAVI) was initially introduced for patients with symptomatic AS but had a very high risk of mortality if surgery was performed [22, 23]. With the progressive feasibility of the performance of TAVI, multiple interventional approaches with feasible outcomes, and statistically better prognosis for patients with intermediate to low surgical risk after TAVI, it became a more feasible option for treatment. However, the time of intervention and strategy for asymptomatic patients are still not clear to date [24,25].

The risk of likely mortality or morbidity for intervention is calculated based on the Society of Thoracic Surgeons Predicted Risk of Mortality (STS-PROM) or the European System for Cardiac Operative Risk Evaluation (EuroSCORE). STS-PROM underestimates and EuroSCORE overestimates the risk. However, STS-PROM is more commonly used in clinical practice to calculate the risk involved [26].

STS scores >15% represent extremely high-risk patients where any intervention, whether surgical or TAVR, has no proven mortality benefit [27]. Patients with an STS score >8% are considered a high-risk cohort, where TAVR should take precedence over SAVR [16]. PARTNER Trials proved that TAVR is non-inferior to SAVR in this cohort [16,17], whereas US Pivotal Trials proved TAVR to be a superior option over SAVR [28]. Patients with STS scores of 4-8% (or EuroSCORE II of 2-10) are considered intermediate-risk patients [29,30]. PARTNER 2 Trial [17], NOTION Trial [31], and OBSERVANT trial [32] have illustrated that TAVR and SAVR have equal long-term all-cause mortality reduction; however, TAVR is also associated with a low peri-interventional complication rate [33]. These results were reconfirmed by a more extensive SURTAVI Trial, which proved the non-inferiority of TAVR over SAVR for the intermediate-risk cohort. Additionally, SAVR was associated with a high incidence of acute renal Injury and atrial fibrillation, and TAVI had higher chances of residual aortic regurgitation and pacemaker implantation [34].

Aortic Valve Replacement Versus Conservative Treatment in Asymptomatic Severe Aortic Stenosis (AVATAR) Trials

Aortic Valve Replacement Versus Conservative Treatment in Asymptomatic Severe Aortic Stenosis (AVATAR) Trials were multicentric, event-driven, intention-to-treat prospective analyses performed to elicit the difference in outcome for early surgical intervention as compared to conservative treatment. Patient enrollment was performed using a negative exercise tolerance test. A total of 157 patients were enrolled in the trial over five years with equal allocation to surgical (n=78) and conservative treatment (n=79) options. Patients were enrolled to enlist a minimum of 35 pre-specified events. The patients were followed up for a median follow-up time of 32 months. The average age of the patients was 67, while the median Society of Thoracic Surgery prognostic score was 1.7%. Of note, 84.7% of patients had AS because of degenerative changes, 14.0% had AS because of the bicuspid aortic valve, and 1.3% had rheumatic heart disease. There was 1.4% operative mortality for the surgical group. There was no statistical significance between two groups with regard to BMI (p=0.59), body surface area (p=0.41), smoking (p=0.67), hypertension (p=0.44) mean systolic pressure (p=0.33), mean diastolic pressure(p=0.33), peripheral artery disease (p=0.80), coronary artery disease (CAD) (p=0.37), BNP levels (p=0.61), total cholesterol (p=0.91), creatinine level (p=0.27), HbA1c (p=0.15), use of β blockers, (p=0.52), ACE inhibitors (p=0.47), calcium channel blockers (p=0.86), diuretics (p=0.48), statins (p=0.22), and antiplatelet agents (p=0.47). Similarly, there was no statistically significant difference in LV end-systolic volume (p=0.96), LV end-diastolic volume (p=0.54), or LVEF (p=0.61). Patients enrolled in the surgical arm had a statistically significant lower incidence of composite all-cause mortality, stroke, myocardial infarction (MI), or heart failure than in the conservative arm (HR: 0.46, 95% CI: 0.23-0.90, p=0.02); however, there was no significant benefit noted with regards to first heart failure hospitalization, thromboembolic complications or major bleeding [35].

Randomized Comparison of Early Surgery Versus Conventional Treatment in Very Severe Aortic Stenosis (RECOVERY) Trial

Almost one-third to half of the patients presenting with AS are asymptomatic at the time of presentation [36,37]. Clinical observation is the current best practice for these asymptomatic patients as the incidence of sudden cardiac death in these patients is about 1% per year, which is not greater than the risk of mortality in the immediate and 30 days post-surgical period and the risk of complication related to aortic valve prosthesis [37,38]. A total of 273 patients having severe AS defined by transvalvular velocity of >4.5 m/s or aortic-valve area <0.75 cm^2^ or >50 mmHg mean transaortic gradient on echocardiogram were enrolled in the trial to compare one-year post-surgical valve replacement versus conservative treatment. Of note, 145 patients completed the enrollment and screening and were assigned to the SAVR or observational group on a 1:1 basis. The mean age of the participants was 64.2 ±9.4 years and 49% were males; 36 (50%) patients assigned to the SAVR group received a biological prosthesis and 36 (50%) received a mechanical valve. There was no mortality reported in the early surgery group. Of the 72 patients assigned to the conservative treatment group, 53% of patients became symptomatic and underwent SAVR (52 patients) and TAVR (one patient). Patients were followed up for four years. The NNT with early surgery to prevent one cardiovascular mortality in four years was 20. When compared to conservative management, patients who received early surgery had a reduced risk of death from cardiovascular causes (HR: 0.09, 95% CI: 0.01-0.67), death from other causes (HR: 0.33, 95% CI: 0.12-0.90), clinical thromboembolic event (HR: 0.30, 95% CI: 0.04-2.31), repeat aortic-valve surgery (HR: 0.19, 95% CI: 0.10-8.00), and hospitalization from heart failure (HR: 0.05, 95% CI: 0.00-1.05) [5]. 

United Kingdom Transcatheter Aortic Valve Implantation (UK TAVI) Trial

The UK TAVI trial was published in 2020, where 913 patients with severe symptomatic AS were enrolled to compare TAVR to SAVR; 458 patients were assigned to TAVR, and 455 were assigned to the SAVR group. The target population for the trial was patients older than 80 years of age with low risk for intervention and patients older than 70 years with intermediate or high surgical risk. It was found that all-cause mortality for patients undergoing TAVR at one year of follow-up was 4.6% as compared to 6.6% in the SAVR group (p=0.23). Additional secondary outcomes included in trials were cardiovascular death (2.8% vs. 3.3% p: 0.69), stroke (5.0% vs. 2.9%; p: 0.13), major bleeding (6.3% vs. 17.1%; p<0.001), permanent pacemaker placement (12.2% vs. 6.6%; p<0.001), vascular complications (4.8% vs. 1.3%, p<0.001) and moderate aortic insufficiency (2.3% vs. 0.6%). The trial concluded that TAVR is non-inferior to SAVR regarding all-cause mortality. Though TAVR was associated with more vascular complications, paravalvular leaks, and permanent pacemaker placement, it had fewer chances of acute bleeding problems and shortened the hospital stay. The risk of stroke was found to be similar between both groups [39].

Nordic Aortic Valve Intervention (NOTION) Trials

The NOTION trial was conducted between 2010 and 2013 to evaluate the difference in the benefit of aortic valve replacement, whether surgical or transcatheter, for patients who are at low risk (2, 12,21). A total of 280 patients were enrolled in the trial, with 145 assigned to undergo TAVI and 135 assigned to SAVR. Three patients from TAVR were crossed over to SAVR for peri-procedural complications and two patients in SAVR could not undergo valve replacement, making the final allocation of 274 patients with 139 in TAVI and 135 in the SAVR group. The mean age of the patients was 79.1 ±4.8 years and the mean STS-PROM score was 3.0% ±1.7%. Patients were followed up annually for eight years after the initial three monthly follow-ups for one year. The extended follow-up concluded that for patients with low surgical risk, there was no significant difference between TAVR and SAVR groups regarding all-cause mortality (51.8% vs. 52.6%, HR: 0.98, 95% CI: 0.71-1.36, p=0.90), stroke (8.3% vs. 9.1%, HR: 0.93, 95% CI: 0.42-2.08, p=0.90), cardiovascular deaths (40.6% vs. 43.6%, HR: 0.93, 95% CI: 0.64-1.34, p=0.93), and MI (6.2% vs. 3.8%, HR: 1.70, 95% CI: 0.57-5.07, p=0.33). Similarly, no significant difference was found between the functional status of patients in either group with extended follow-up. Based on the extent of the peri-ventricular leak (PVL) following the TAVI three months post-procedure, there was no difference in risk of mortality when comparing those with moderate-to-severe PVL to those with trace/no PVL (13.8% vs. 76.5%, p=0.53) (all-cause mortality: 55.0% vs. 48.3%, p=0.53) [31].

Extended follow-up outcomes of TAVR in patients at low surgical risks

Over the last decade, multiple trials have demonstrated the non-inferiority of TAVR over SAVR for the high- and intermediate-risk adult population [5,23]. Five years of follow-up on patients from these trials have shown sustained clinical effects and durable valve performance. PARTNER 3 trials have demonstrated the safety of the SAPIEN 3 transcatheter valve in low-risk patients, showing a 46% reduction in the one-year mortality following TAVR as compared to SAVR (p=0.001) [40]. Leon et al. conducted a two-year follow-up trial on PARTNER 3 trial that enrolled patients to analyze the durability of TAVR using the SAPIEN 3 valve for low-risk patients [17]. A total of 950 patients were included, of which 496 underwent TAVR and 454 underwent SAVR. The mean age of the patients was 73 years, and the mean STS-PROM score was 1.9%. At two years of follow-up, the composite death rate from all causes was found to be 11.5% in the TAVR cohort and 17.4% in the SAVR cohort (HR: 0.63; 95% CI: 1.13-2.23, p=0.008). Similarly, mean event-free survival at two years was improved with TAVR as compared to SAVR (670 days vs. 622 days, p<0.001). In the sub-analysis, the event rates for TAVR vs. SAVR for all-cause mortality were 2.4% vs. 3.2% (HR: 0.75, 95% CI: 0.35-1.63, p=0.47); for stroke were 2.4% vs. 3.2% (HR: 0.66, 95% CI: 0.31-1.40, p=0.28); and for rehospitalization were 8.5% vs. 12.5% (HR: 0.67, 95% CI: 0.45-1.00, p=0.046). At the two-year follow-up, TAVR was found to be associated with an increased likelihood of death than SAVR (seven vs. three), stroke (six vs. one), and a similar number of rehospitalization (10 vs. 8). The combined endpoint of either death or disabling stroke at two years was 3.0% in TAVR vs. 3.8% in SAVR. (HR: 0.77, 95% CI: 0.39-1.55, p=0.47). New York Heart Association (NYHA) and Kansas City Cardiomyopathy Questionnaire (KCCQ-OD) were used to analyze the difference in the functional status between both groups. There was a substantial improvement in QOL at two years regardless of the approach used from the baseline. TAVR was found to be superior to SAVR at one-month, one-year, and two-year follow-up regarding the QOL index. There was a small difference between one- and two-year follow-ups regarding hemodynamic findings and LV function changes. The mean gradient was higher after TAVR vs. SAVR at the two-year follow-up (13.6 ±5.53 vs. 11.8 ±4.82, p=0.06), whereas the effective orifice area was similar in TAVR vs. SAVR (1.7 ±0.37 vs. 1.7 ±0.42, p=0.34). These findings suggest that primary endpoints decreased by 37% in the TAVR group; however, between years one and two, the rates of death and disabling stroke were more than in SAVR, such that at two years, the difference between the two groups was not significant. TAVR was associated with more frequent valve thrombosis and increased valve gradient by 54%. Though there was a significant improvement in the QOL scores with TAVR, there was no major improvement in echocardiographic findings in TAVR at two years of follow-up [17].

Impact of end-stage renal disease (ESRD) on the choice of treatment

Patients with end-stage renal disease (ESRD) are a special high-risk group of patients who have increased mortality risk and poor outcomes following SAVR as compared to the normal population [41,42,43,44]. Hence, balloon aortic valvoplasty (BAV) has been used as a palliative option in these patients. Similarly, due to increased mortality risk, they are also excluded from major trials [45]. A meta-analysis was conducted by Condado et al. to compile baseline characteristics, risks, and benefits in patients with ESRD undergoing aortic valve intervention [46]. A total of 85 patients were involved in various trials from 2007 to 2015; 28.4% had BAV, 35.3% had TAVR, and 35.3% had SAVR. Patients who underwent BAV and TAVR, when compared to patients who underwent SAVR, were older (74 vs. 71 vs. 63 years, p=0.02), had history of previous cerebrovascular accident (CVA) (12.0% vs. 30.0% vs. 3.3%, p=0.01), decreased functional status (NYHA class III/IV 100% vs. 93.3% vs. 76.7%, p=0.01), and had higher STS scores (13.5% vs. 13.5% vs. 8.6%, p=0.08). There was no difference in the three groups regarding the history of chronic obstructive pulmonary disease, hypertension, and percutaneous coronary intervention (PCI). Despite inherently greater contrast use, increased fluoroscopy dose, and fluoroscopy time with TAVR, there was no statistical benefit with regard to procedural success rate (p=0.10), early safety outcome (p=0.95), or 30-day outcomes. However, patients who underwent BAV had significantly higher one-year mortality when compared to TAVR or SAVR (87.0% vs. 32.0% vs. 36.7%, p<0.001), though there was no statistically significant difference in operative and 30-day mortality among the three groups. There was no statistically significant difference in one year between the groups with regard to the type and duration of dialysis. The higher mortality at one year noted in the BAV group was attributed to the higher proportion of patients with symptomatic disease. It was also determined that patients undergoing dialysis who underwent SAVR and TAVR had an absolute risk reduction (ARR) of 55% and 51% respectively as compared to the 20% ARR noted in the general population in the PARTNER I Trial [46]. 

Impact of AKI over CKD in patients with aortic stenosis

Chronic kidney disease (CKD) is a common comorbid finding in a patient with AS, with incidence reaching up to 75% in patients seeking valve replacement of any kind [47]. Preoperative CKD is an independent risk factor for AKI following SAVR or TAVR [48,49,50]. Patients who develop AKI following valve replacement have a significantly higher length of inpatient stay and higher 30-day and one-year mortality [51,52]. PARTNER Trials revealed no significant difference in the risk of development of AKI following SAVR or TAVR [53]. Similar results were reported by Thongprayoon et al. [54]. Contrary to these findings, analysis conducted by Bagur et al. [55] and D'Errigo et al. [56] found that the incidence of development of AKI following TAVR was significantly lower than those undergoing SAVR [(9.2% vs. 25.9%, p:0.001), (35.8 vs. 48.9%, p:0.04) respectively)]. A retrospective study was conducted by Catalano et al. on 813 patients having AS and CKD stage III or worse, out of which 406 patients underwent SAVR and 407 patients underwent TAVR. Patients requiring TAVR were comparatively older, leaner, had dyslipidemia, and had a higher preoperative ejection fraction than those undergoing SAVR. There was no significant difference between both groups with regard to gender and comorbidities including diabetes, hypertension, or heart failure. However, patients undergoing SAVR had significantly lower STS-PROM scores (median: 5.0% vs. 7.7%, p<0.001). The difference in 30-day mortality in the subgroup was not significant between the two groups (OR: 2.38, 95% CI: 0.85-6.64, p<0.10). Patients undergoing TAVR were found to be significantly less likely to develop AKI per RIFLE classification than those undergoing SAVR (11.78% vs. 38.30%) [55]. Patients undergoing SAVR were more likely to require postoperative dialysis as compared to TAVR patients (OR: 4.55, 95% CI: 1.29-15.99, p<0.018). After the stratification of data based on the STS-PROM scores, there was no significant difference between the groups. SAVR was associated with an increased risk of post-procedure AKI (OR: 4.75, 95% CI: 3.04-7.41; p<0.01) and dialysis (OR: 5.31, 95% CI: 1.31-21.54; p<0.02). There was no significant association between bypass time for patients undergoing SAVR and AKI (OR: 1.00, 95% CI: 1.00, p=0.20). Similarly, there was no significant association in the amount of contrast used for TAVR and AKI (OR: 1.00, 95% CI: 0.99-1.01, p=0.68). The median contrast used for TAVR was found to be 69 cc. It was also illustrated that the lowest eGFR was reached on day two after TAVR vs. day three for SAVR [57].

Concomitant PCI and TAVR

CAD is a common finding among patients with AS, as both have common risk factors [58,59]. FRANCE 2 Registry of TAVR patients showed that 47.9% of the patients had CAD [60]. Similarly, 74.9% of the patients with AS had CAD in the PARTNER Trial [61]. Performing SAVR with coronary artery bypass surgery (CABG) has been the standard of care [62]. In the patients undergoing TAVR, PCI is either performed concurrently or staged before the procedure due to the inherent difficulty of performing PCI following placement of the prosthetic valve frame, interfering with the guiding catheter [63]. A systematic review conducted by Yang et al. in 2017 showed that all-cause mortality at 30 days was 13.2% for concomitant PCI and TAVR, whereas, in staged PCI and TAVR, it was 11.3% (OR: 1.47, 95% CI: 0.47-4.62, p=0.51). Similarly, the incidence of renal failure was also not statistically significant between both groups (OR: 3.22, 95% CI: 0.61-17.12, p=0.17). Additionally, there was no statistically significant difference between the two groups with regard to peri-procedural MI (OR: 1.44, 95% CI 0.12-16.94, p=0.77), life-threatening bleeding (OR: 0.45, 95% CI: 0.11-1.87, p=0.27) and major stroke (OR: 3.41, 95% CI: 0.16-74.2, p=0.44). These findings are in concordance with those of Penkalla et al., where it was demonstrated that 30-day all-cause mortality was similar in patients who underwent PCI and TAVR simultaneously because of significant CAD, and those who had only TAVR due to the absence of CAD [64]. It has been found that either concomitant PCI and TAVR and staged TAVR and PCI use almost similar amount of contrast medium, 343 ±126 ml vs. 330 ±140 ml [65]; therefore, there was no significant difference in the incidence of contrast-induced nephropathy in either group as well [64,66,67]. 

Effect of chronic thrombocytopenia on TAVR

Thrombocytopenia is reported at baseline in 20-40% of the patient undergoing TAVR [68,69]. Fugar et al. [70] conducted a meta-analysis of 60990 cases of TAVR using the NIS database. With the exclusion of peri-procedural thrombocytopenia, 4300 patients (7.3%) were found to have chronic thrombocytopenia (CTP). Patients with thrombocytopenia were males and had a higher prevalence of comorbid conditions including anemia, congestive heart failure, liver disease, peripheral vascular disease, implantable cardioverter defibrillator (ICD) placement, smoking, pacemaker placement, chronic renal failure, and neurological disorder (p<0.05). Using a propensity matched cohort of 4300 patient not having CTP, patients with CTP were found to have a higher in-hospital mortality (OR: 3.21, 95% CI: 2.77-3.72, p<0.001), increased risk of post-procedural hemorrhage (OR: 1.91, 95% CI: 1.43-2.55, p<0.001) platelets transfusion (OR: 3.47, 95% CI: 2.60-4.63, p<0.001) and RBCs transfusion (OR: 1.30, 95% CI: 1.12-1.52, p=0.0.001), vascular complications (OR: 1.81, 95% CI: 1.41-2.34, p<0.001), cardiac tamponade requiring intervention (OR: 9.03, 95% CI: 4.87-16.73, p<0.001) and AKI (OR: 3.54, 95% CI: 2.99-4.45, p<0.001), whereas the risk of acute MI (OR: 0.93, 95% CI: 0.61-1.41, p=0.731), and post-procedural ischemic cerebral event (OR: 1.35, 95% CI: 0.94-1.95, p=0.105) were same between both groups. Platelet transfusion was also found to be associated with increased in-hospital mortality and vascular complication including MI and CVA. To determine platelets as an independent risk factor, the backward logistic regression model was performed. Predictors of increased mortality were found to be diabetes (OR: 1.63, 95% CI: 1.18-2.25, p=0.003), chronic weight loss (OR: 3.73, 95% CI: 1.32-10.59, p=0.013), CAD (OR: 3.73, 95% CI: 1.59-3.34, p<0.001), cardiac tamponade (OR: 9.58, 95% CI: 4.91-18.7, p<0.001), acute CVA (OR: 3.69, 95% CI: 2.23-6.120, p<0.001), and AKI (OR: 3.08, 95% CI: 3.073-4.28, p<0.001).

Patients with CTP have significantly higher resource utilization. They were also found to have longer hospital stays (median of six vs. five, p<0.001), and higher cost of management (median $54773 vs. $49801, p<0.001). Patients with CTP were more likely to be discharged to skilled facilities (34.1% vs. 27.6%, p<0.001). The cost of care was significantly higher if the patient developed a perioperative complication (incremental cost: $6920), acute ischemic CVA (incremental cost: $21967), or vascular complication (incremental cost: 24,294). However, there was no significant cost increment in patients who required blood transfusions, developed acute MI or AKI, or post-procedural hemorrhage [69]. Similar results were reported by Flaherty et al., who analyzed a case series of 90 patients undergoing TAVR having severe symptomatic thrombocytopenia. It was reported that preexisting CTP was associated with major thrombocytopenia (<100000 cell/ml) and >50% fall from baseline), which in turn leads to a higher risk of major bleeding (OR: 3.18, 95% CI: 1.33-5.42) and vascular complications (OR: 2.78, 95% CI: 1.58-3.82) [68]. Similar results were reported in a retrospective study of 732 subjects undergoing TAVR with severe symptomatic AS [68]. CTP was found to be an independent risk factor for 30-day (HR: 13.18, 95% CI: 4.49-38.64, p<0.001) and one-year all-cause mortality (HR: 5.90, 95% CI: 2.68-13.02, p<0.001) [70].

SAVR in asymptomatic patients

American Heart Association, American College of Cardiology, European Society of Cardiology, and European Association of Cardio-Thoracic Surgery recommend surgical intervention for asymptomatic patients only if they have LVEF <50%, have demonstrable symptoms on the exercise stress test, and are undergoing cardiac surgery for other reasons [71]. 

Medtronic Evolut Transcatheter Aortic Valve Replacement in Low-risk Patients (Evolut Low Risk) Trial 

To date, Medtronic Evolut Trial is one of the two trials that have compared the impact of TAVR in patients with asymptomatic AS. Evolut Trial was a randomized multi-centric event, which evaluated the safety of CoreValve™, Evolut™ R, and Evolut™ PRO valves, in low-surgical risk patients. The trial enrolled class 1 NYHA patients having low STS-PROM scores to compare the clinical outcomes, QOL index, and echocardiographic findings. The Evolut Trial is an ongoing clinical trial, where 1468 participants were enrolled and are currently being followed up. Patients were enrolled based on whether they had severe AS with mean gradient ≥60 mmHg, maximal aortic velocity ≥5.0 m/s, severe AS with less than 50% LVEF, or those with reduced exercise tolerance; the choice of treatment was assigned based on the patient`s discretion. The trial found comparable results between groups of patients undergoing SAVR and TAVR with regard to composite all-cause mortality and stroke (6.3% vs. 1.3%, p=0.11) at 12 months based on `as-treated analysis. A sub-analysis of 138 participants who were asymptomatic at the time of enrollment has been published to present one-year follow-up results for the cohort. Patients who underwent TAVR showed considerably low mean aortic valve gradient (8.1 ±3.2 mmHg vs. 10.8 ±3.8 mmHg, p<0.001), larger effective orifice area (2.3 ±0.6 cm^2^ vs. 1.9 ±0.6 cm^2^, p=0.001) and improvement in KCCQ at 30 days from baseline (Δ12.1 ±23.6 vs. Δ2.2 ±20.3, p<0.001). Regardless of the type of intervention used, there was a significant improvement in the QOL index from baseline in all patients. Though the cerebral embolic protection device was not used in the trial for patients undergoing TAVR, previous trials have shown that the use of a protective device helps reduce the stroke rate significantly in those undergoing TAVR [72]. The findings of the two-year follow-up were published in May 2021, where it was found that TAVR continues to show improved safety at two-year follow-up. Additionally, TAVR shows improved hemodynamic performance, improved rates of death, heart failure hospitalization, and prosthesis-patient mismatch. Valve thrombosis rates remained stable over two years. Major limitations found in the study designs include the small number of patients enrolled and the exclusion of patients with the bicuspid aortic valve and extensive left ventricular outflow tract (LVOT) calcification. Additionally, extended follow-up of the enrolled patients has been advised to define long-term prognostic value [73].

Early TAVR Trial

The Early TAVR Trial was conducted to prove the safety and efficacy of Edwards SAPIEN 3/SAPIEN 3 Ultra Trans-catheter Heart Valve in asymptomatic AS patients with low surgical risk as compared to clinical surveillance. The study is an ongoing multi-centric trial, where 901 participants were recruited using parallel randomized assignments to be assigned to either of the groups. The recruitment was started in June 2017, and currently, the recruited patients are being followed up to complete the trial protocol that is assigned a two-year follow-up period. The primary outcome of the study is to document all-cause death and two years' risk of hospitalization for stroke and cardiovascular reasons. Additional outcomes that are being considered as secondary outcomes are the Kansas City Cardiomyopathy Questionnaire (KCCQ) score improvement, improvement in echocardiographic findings, documented improvement in LV health including improvement in LVEF, the incidence of new-onset atrial fibrillation, and disabling stroke or death. Patients were recruited using results of exercise stress tests and echocardiography to document asymptomatic disease and LVEF ≥50%. Patients with severe aortic valve disease or decompensated LV function for any concomitant disease were excluded from the trials. Though the initial results shared in press media are said to be positive favoring TAVR over conservative management, the official results and recommendations are still awaited [74].

## Conclusions

Convincing a patient who is asymptomatic to undergo intervention is a quite challenging task. Immediate and intermediate-term complications of SAVR or TAVR must be taken into consideration when making the referral for intervention. Early valve replacement decreases mortality risk by half regardless of whether TAVR or SAVR is used. This benefit is not confounded by BMI, body surface area, smoking, hypertension, mean systolic and diastolic volume, LVEF, presence of peripheral or coronary artery disease, pro-BNP levels, cholesterol level, creatinine levels, HbA1c, use of beta-blockers, ACE inhibitors, statins, or diuretics when compared to conservative treatment. TAVR has been associated with a significantly lower chance of perioperative complications, and better QOL as compared to SAVR. Regardless of the type of dialysis, patients with ESRD show comparable benefits in terms of success rate, early safety, or 30-day mortality after SAVR or TAVR. Patients with ESRD were found to have a 50% reduction in ARR in mortality benefits after intervention as compared to a 20% ARR with intervention in the normal population. TAVR is also preferred in ESRD for a lower risk of AKI as compared to SAVR. In the presence of CAD, TAVR has comparable benefits whether concomitant or staged PCI is performed with TAVR. CTP must be corrected before intervention to decrease mortality risk and incidence of complications after the procedure.

Evolut Trial's sub-analysis has shown improvement in the QOL index, trans-valvular gradient, and valvular orifice area after TAVR. Though similar results are being reported for the Early TAVR Trial, official results are still awaited. However, further randomized clinical trials are required to stratify the benefits of TAVR over conservative management, to elicit the mortality benefit of TAVR in patients with asymptomatic AS, and to statistically delineate the population characteristics that will make TAVR a preferred intervention for asymptomatic AS patients.
